# Optimization of Left Ventricle Pace Maker Location Using Echo-Based Fluid-Structure Interaction Models

**DOI:** 10.3389/fphys.2022.843421

**Published:** 2022-02-17

**Authors:** Longling Fan, Jing Yao, Liang Wang, Di Xu, Dalin Tang

**Affiliations:** ^1^Faculty of Science, Kunming University of Science and Technology, Kunming, China; ^2^School of Mathematics, Southeast University, Nanjing, China; ^3^Department of Ultrasound Medicine, Affiliated Drum Tower Hospital of Nanjing University Medical School, Nanjing, China; ^4^School of Biological Science and Medical Engineering, Southeast University, Nanjing, China; ^5^Department of Cardiology, First Affiliated Hospital of Nanjing Medical University, Nanjing, China; ^6^Mathematical Sciences Department, Worcester Polytechnic Institute, Worcester, MA, United States

**Keywords:** fluid-structure interaction model, pacemaker electrical conduction, fluid dynamic, ventricle material properties, ventricle mechanics

## Abstract

**Introduction:**

Cardiac pacing has been an effective treatment in the management of patients with bradyarrhythmia and tachyarrhythmia. Different pacemaker location has different responses, and pacemaker effectiveness to each individual can also be different. A novel image-based ventricle animal modeling approach was proposed to optimize ventricular pacemaker site for better cardiac outcome.

**Method:**

One health female adult pig (weight 42.5 kg) was used to make a pacing animal model with different ventricle pacing locations. Ventricle surface electric signal, blood pressure and echo image were acquired 15 min after the pacemaker was implanted. Echo-based left ventricle fluid-structure interaction models were constructed to perform ventricle function analysis and investigate impact of pacemaker location on cardiac outcome. With the measured electric signal map from the pig associated with the actual pacemaker site, electric potential conduction of myocardium was modeled by material stiffening and softening in our model, with stiffening simulating contraction and softening simulating relaxation. Ventricle model without pacemaker (NP model) and three ventricle models with the following pacemaker locations were simulated: right ventricular apex (RVA model), posterior interventricular septum (PIVS model) and right ventricular outflow tract (RVOT model). Since higher peak flow velocity, flow shear stress (FSS), ventricle stress and strain are linked to better cardiac function, those data were collected for model comparisons.

**Results:**

At the peak of filling, velocity magnitude, FSS, stress and strain for RVOT and PIVS models were 13%, 45%, 18%, 13% and 5%, 30%, 10%, 5% higher than NP model, respectively. At the peak of ejection, velocity magnitude, FSS, stress and strain for RVOT and PIVS models were 50%, 44%, 54%, 59% and 23%, 36%, 39%, 53% higher than NP model, respectively. RVA model had lower velocity, FSS, stress and strain than NP model. RVOT model had higher peak flow velocity and stress/strain than PIVS model. It indicated RVOT pacemaker site may be the best location.

**Conclusion:**

This preliminary study indicated that RVOT model had the best performance among the four models compared. This modeling approach could be used as “virtual surgery” to try various pacemaker locations and avoid risky and dangerous surgical experiments on real patients.

## Introduction

Rapid development of cardiac pacing has recently become the only effective treatment for slow cardiac arrhythmia (Glikson et al., 2021). Between 2004 and 2014, 2.9 million patients received permanent pacemakers in the United States (Mohamed et al., 2019). China Ministry of Health Online Registration indicated that pacemaker implants were placed in 82779 patients in 2018, and the number has been increasing year by year (Liu, 2019). Right ventricular apex (RVA) has been the conventional location for pacemaker lead placement. However, RVA is a non-physiological agonist site that causes the electrical and mechanical origin and distribution patterns of the heart to be opposite to normal sinus rhythm, resulting in hemodynamic abnormalities and tissue remodeling (Kapa et al., 2010). The review by Tops et al. (2009) provided a contemporary overview of the available evidence on the detrimental effects of RVA pacing. Furthermore, clinical trials have shown that RVA pacing can cause electro-mechanical contraction of the left and right ventricles to be asynchronous. Long-term RVA pacing can cause cardiac histologic and electrical remodeling, abnormal myocardial contractile pattern, hemodynamic disorder, and ultimately cardiac insufficiency, there is also the risk of atrial fibrillation (AF) and heart failure (HF), which increases the rate of hospitalization and mortality (Manolis et al., 2008; Miranda et al., 2012; Alhous et al., 2015). So optimization of right ventricular pacing site becomes an important object of pacing electrophysiology. In recent years, the concept of physiological pacing has been proposed in the field of electrophysiological (Das and Kahali, 2016; Vijayaraman et al., 2017; Liu et al., 2021), and the study of the selection of pacing sites has received great attention (Coppola et al., 2015; Carpio et al., 2019; Zhu et al., 2020). The physiological pacing sites currently studied include the His bundle or para-His bundle (Kronborg et al., 2014; Sharma et al., 2020), the right ventricular inflow tract septum (Tsujii et al., 2016), the right ventricular outflow tract (RVOT) (Da Costa et al., 2013; Yao et al., 2013; Zou et al., 2015), Left bundle branch area (Das et al., 2020; Michalik et al., 2021), and so on. Here His indicates His bundle which is a collection of heart muscle cells specialized for electrical conduction. As part of the electrical conduction system of the heart, it transmits the electrical impulses from the atrioventricular node (located between the atria and the ventricles) to the point of the apex of the fascicular branches via the bundle branches. Studies have shown that the above-mentioned pacing site can increase cardiac output and improve cardiac function compared with conventional RVA pacing, but these sites have advantages and disadvantages in terms of operation technique, stability, pacing threshold, etc. (Erdogan and Hunuk, 2010; Singh et al., 2015; Alberti et al., 2017). Moreover, Individual differences in heart disease such as myocardial infarction, cardiomyopathy, valvular disease, etc., myocardial contraction/diastolic function, and myocardial thickness can influence the response of the pacemaker, thus could produce different cardiac functional responses.

The analysis of electrical, mechanical and flow fields in patients with pacemaker implantation has been a hot topic in the field of pacing electrophysiology, and most of these studies focus on electro-mechanical coupling. Usyk et al. (2012) established a three-dimensional biventricular electrical complex model that reflects the local myocardial mechanics of the ventricle, and completely simulated the entire cycle of the heart. Zhu et al. (2001) based on the constructed cardiac electrophysiological model and proposed an algorithm for calculating ECG based on single-cell action potential. Gurev et al. (2010) established a three-dimensional electrophysiological model of the rabbit ventricle and used it to analyze the three-dimensional distribution of ventricular fibril contraction delay and its dependence on loading conditions. Recent advances in computational modeling, methods and computer technology have made it possible for computer-simulated procedures to be used in clinical decision-making for specific patients. The feasibility to integrate computational modeling with clinical investigations in a clinical environment and to guide therapeutic treatment of cardiac arrhythmia and heart failure in real time for individual patients has been previously demonstrated (Chen et al., 2017). Lee et al. (2018) focus on the contribution of electrophysiology, mechanics, and circulatory computer models of the heart to understanding cardiac resynchronization therapy response. In our previous studies, we introduced patient-specific cardiac magnetic resonance (CMR)-based left ventricular/right ventricular (LV/RV) models with fluid-structure interactions (FSI) with various surgical design and potential applications (Tang et al., 2010, 2011, 2016; Huang et al., 2021). Echo-based 3D LV FSI models were introduced to perform ventricle mechanical analysis and investigate flow behaviors (Fan et al., 2018).

This paper will integrate echocardiography images, propagating dynamic electric potential on ventricle surface induced by pacemaker, and computational models with fluid-structure interactions (FSI) to perform myocardial function and intra-cardiac flow assessment. Ventricle model without pacemaker (NP model) and three ventricle models with the following pacemaker locations were simulated: right ventricular apex (RVA model), posterior interventricular septum (PIVS model) and right ventricular outflow tract (RVOT model). The models will be used to evaluate the impact of pacemaker locations and optimize its placement.

## Materials and Methods

### Animal Model Preparation

**FIGURE 1:**
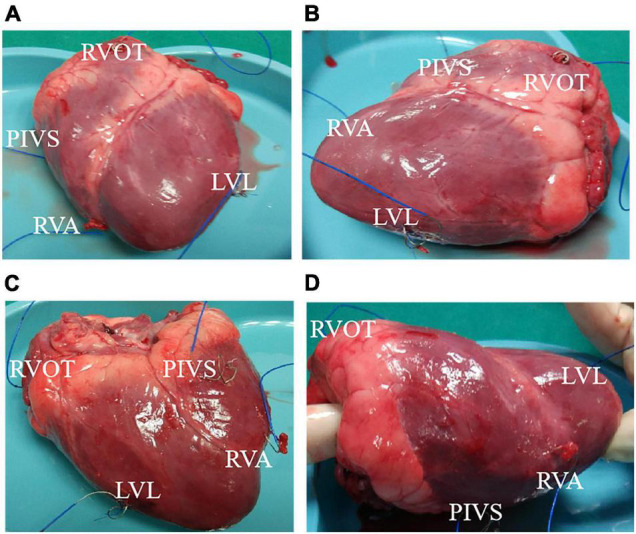
The position of the pacemaker lead in the heart. **(A)** Front view of heart. **(B)** Left side view of heart. **(C)** Back view of heart. **(D)** Right side view of heart. RVA, right ventricular apex; PIVS, posterior interventricular septum; RVOT, right ventricular outflow tract; LVL, left ventricular lateral wall.

The animal study were approved by the Ethics Committee of the First Affiliated Hospital of Nanjing Medical University and all applicable institutional and governmental regulations concerning the ethical use of animals were followed. One health female adult pig weight 42.5kg was used to make pacing animal model under different ventricular pacing location. Health status was assessed before undergoing the experimental procedures. The pig was intubated and mechanically ventilated, and anesthesia was maintained using isoflurane. A 5-7cm left anterolateral thoracotomy was made in the fourth left intercostal space. After opening the chest, a transverse incision of approximately 2-3cm was made in the pericardial sac. Parietal pericardium was made with cotton 2-0 stitches to expose the ventricle and the left atrium. Epicardial pacing leads were fixed to following locations: right atrial appendage (RAA), right ventricular apex (RVA), posterior interventricular septum (PIVS), and right ventricular outflow tract (RVOT), and left ventricular lateral wall (LVL). The tip of RAA lead was connected to atrial terminals of Medtronic Dual Chamber Temporary cardiac pacemaker. Then RVA, PIVS, RVOT, and LVL lead was connected to the Ventricular pacing, respectively. Figure 1 shows the positions of the pacemaker leads in the heart.

### 3D Echo Data Acquisition

**FIGURE 2:**
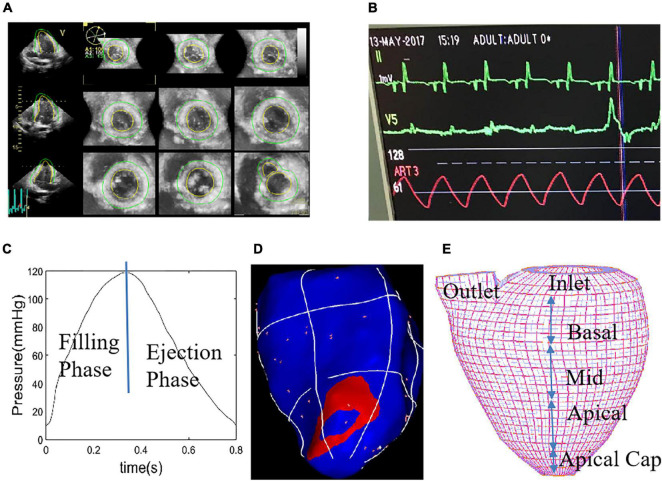
Echo image of right ventricular apex (RVA) pacemaker, pressure profile, electric signal mapping and re-constructed left ventricular (LV) geometry with mesh. **(A)** End-Systolic Echo image, RVA. **(B)** Recorded LV blood pressure profile. **(C)** Imposed LV blood pressure profile. **(D)** Electric signal mapping, RVA model. **(E)** Re-constructed LV geometry with mesh which was used to divide LV into small regions to specify tissue stiffness variations corresponding to electric signal propagation.

Electric potential data recording and echocardiographic image acquisition were started 15 min after stabilization of pacing model. Real-time three-dimensional full-volume image of the apical 4-chamber view was obtained using an ultrasound machine (E9, GE Mechanical Systems, Milwaukee, Wisconsin), with TEE probe 6VT-D attaching to LV apex directly. Electrophysiological recorder records body surface 12-lead electrocardiogram and intracardiac electrogram. Meantime, the pressure gauge catheter was connected to the Medtronic Lifpark12 monitor. The left ventricular pressure curve was measured before and during the time period when the pacemaker was implanted. Table 1 gives basic information including ventricular pacing location, pressure and volume data. We obtained the relationship between the different pacing interventions and the myocardial material function by analyzing the ultrasound images, volume data and cardiac chamber pressure data, determining the function of the myocardial analysis material corresponding to the specific pacing mode, and establishing the left ventricle under different pacing interventions. Three-dimensional FSI models were used to obtain left ventricle stress and strain, and flow velocity and shear stress in the heart chamber. Furthermore, the differences in these variables under different pacing models were analyzed to infer the optimal pacing model for optimal pacemaker implantation. Figure 2 shows the *in vivo* Echo image, pressure curve and electrical signal conduction map, and ventricular partitioning under RVA pacing intervention. The compartment partitions were used to set material parameters to simulate the conduction of electrical signals.

**TABLE 1:** Ventricular pacing location and volume data.

Pacemaker location	NP		RVA		RVOT		PIVS	
Pressure (mmHg)	Min = 9	Max = 102	Min = 10	Max = 119	Min = 8	Max = 90	Min = 7	Max = 83
Echo Vol (ml)	Min = 26	Max = 54	Min = 27	Max = 55	Min = 25	Max = 53	Min = 24	Max = 50
Echo EF (%)	51.85		50.91		52.83		52.00	
Model Vol (ml)	Min = 25.96	Max = 54.01	Min = 27.07	Max = 54.96	Min = 25.02	Max = 53.04	Min = 24.01	Max = 49.98
Model EF (%)	51.93		50.75		52.83		51.96	

### The Fluid-Structure Interaction Model of Left Ventricular

Blood flow in the left ventricle was assumed to be laminar, Newtonian, viscous and incompressible. The Navier-Stokes equations with arbitrary Lagrangian-Eulerian (ALE) formulation were used as the governing equations. To simplify the computational model, the cardiac cycle was split into two phases: (a) the filling phase (diastole) when the inlet was open, inlet blood pressure was prescribed (Figure 2C), blood flows into the LV, and the outlet was closed (by setting flow velocity to zero); (b) The ejection phase (systole) when inlet was closed, outlet was open, outlet pressure was prescribed, and blood was ejected out of the LV. Pressure conditions were prescribed at the mitral (inlet) and aortic (outlet) valves. When the inlet or outlet were closed, flow velocity was set to zero and pressure was left unspecified. When the inlet or outlet was open, blood pressure was prescribed and flow velocity was calculated by ADINA. No-slip boundary conditions and natural force boundary conditions were specified at all interfaces to couple fluid and structure models together (Tang et al., 2010, 2016). Navier-Stokes equation in ALE coordinate system and boundary conditions for fluid model were given:


(1) $$\rho \,\left(\partial \text{u}/\partial \text{t}+\left(\left(\text{u}-{\text{u}}_{g}\right)\cdot \nabla \right)\,\text{u}\right)=-\nabla \text{p}+\mu \,\nabla^{2}\text{u}$$

(2) ∇⋅u=0

(3) $${u|}_{\Gamma }=\left(\partial x/\partial t\right)$$

(4) $${P|}_{i\,n\,l\,e\,t}=p_{i\,n}\,\left(t\right),{\partial u/\partial n|}_{i\,n\,l\,e\,t}=0,{u|}_{o\,u\,t\,l\,e\,t}=0,\left(\mathrm{filling}\,\mathrm{phase}\right)$$

(5) $${P|}_{o\,u\,t\,l\,e\,t}=p_{o\,u\,t}\,\left(t\right),{\partial u/\partial n|}_{o\,u\,t\,l\,e\,t}=0,{u|}_{i\,n\,l\,e\,t}=0,\left(\mathrm{ejection}\,\mathrm{phase}\right)$$

(6) $${\sigma_{i\,j}\cdot n_{j}|}_{o\,u\,t\,w\,a\,l\,l}=0$$

(7) $${\sigma_{i\,j}^{r}\cdot n_{j}^{r}|}_{i\,n\,t\,e\,r\,f\,a\,c\,e}={\sigma_{i\,j}^{s}\cdot n_{j}^{s}|}_{i\,n\,t\,e\,r\,f\,a\,c\,e}$$

where ***u*** and ***p*** are flow velocity and pressure, ***u*_*g*_** is mesh velocity, μ is the viscosity of blood. Γ stands for LV inner wall, *f*_∙,j_ stands for derivative of *f* with respect to the *j*th variable (or time t), σ_r_ and σ_s_ are fluid and structure stress tensors, and **n**^r^ and **n**^s^ are their outward normal directions, respectively.

The ventricle material tissue was assumed to be hyperelastic, anisotropic, homogeneous and nearly incompressible. The governing equations for the LV structure model were:


(8) $$\rho v_{i,t\,t}=\sigma_{i\,j}{,}_{j},i,j=1,2,3;\mathrm{sum}\mathrm{over}j,$$

(9) $$\varepsilon_{i\,j}=\frac{1}{2}\,\left(v_{i,j}+v_{j,i}+v_{\alpha ,i}\,v_{\alpha ,j}\right),i,j,\alpha =1,2,3,$$

where **σ** is the stress tensor, **ε** is the strain tensor, ***v*** is displacement, and ρ is material density. The normal stress was assumed to be zero on the outer (epicardial) LV surface and equal to the normal stress imposed by fluid forces on the inner (endocardial) LV surface as specified by Eq. (7). Fluid and structure were coupled through their interfaces. Fluid-structure interaction must satisfy the traction balance and compatibility conditions for displacement and velocity.

The nonlinear Mooney-Rivlin model was used to describe the nonlinear anisotropic material properties. The strain energy function for the anisotropic modified Mooney-Rivlin model is given:


(10) $$W=c_{1}\,\left(I_{1}-3\right)+c_{2}\,\left(I_{2}-3\right)+D_{1}\,\left[e\,x\,p\,\left(D_{2}\,\left(I_{1}-3\right)\right)-1\right]+\frac{K_{1}}{K_{2}}\text{[}exp{\left(I_{4}-1\right)}^{2}-1]$$

where *I*_1_ and *I*_2_ are the first and second strain invariants given by,


(11) $$I_{1}=\sum C_{i\,i},I_{2}=\frac{1}{2}\,\left(I_{1}^{2}-C_{i\,j}\,C_{i\,j}\right),I_{4}=C_{i\,j}\,{\left(n_{f}\right)}_{i}\,{\left(n_{f}\right)}_{j}$$

**FIGURE 3:**
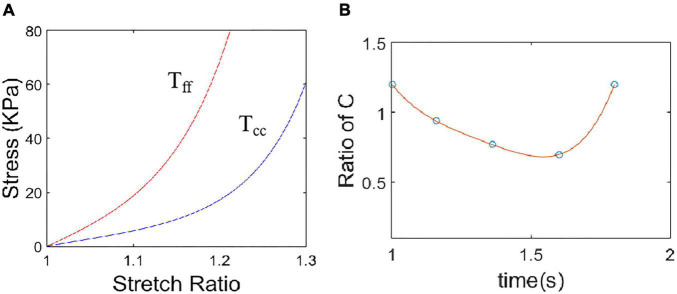
Material stress-stretch curves for a baseline model with right ventricular apex (RVA) pacemaker. **(A)** Baseline material stress-stretch curves, T_ff_: stress in fiber direction, T_cc_: stress in circumferential direction of the fiber. **(B)** Stiffness variation in a cardiac cycle for a sample region of the model with RVA pacemaker. Stiffness variation was realized by adjust C values in Eq. (12).

C = [C_*ij*_] = X^T^X is the right Cauchy-Green deformation tensor, X = [X_ij_] = [∂x_i_/∂a_j_], (x_i_) is the current position, (a_i_) is the original position, **n**_f_ is the fiber direction, c_i_, D_i_ and K_i_ are material parameters chosen to match experimental measurements (Fan et al., 2018; Lee et al., 2018). With parameters properly chosen, it was shown that stress-strain curves derived from Eq. (10) agreed very well with the stress-strain curves from the anisotropic (transversely isotropic) strain-energy function with respect to the local fiber direction given in McCulloch et al. (1992):


(12) $$W=\frac{C}{2}\,\left(e^{Q}-1\right)$$

(13) $$Q=b_{1}\,E_{f\,f}^{2}+b_{2}\,\left(E_{c\,c}^{2}+E_{r\,r}^{2}+E_{c\,r}^{2}+E_{r\,c}^{2}\right)+b_{3}\,\left(E_{f\,c}^{2}+E_{c\,f}^{2}+E_{f\,r}^{2}+E_{r\,f}^{2}\right)$$

where *E*_ff_ is fiber strain, *E*_cc_ is cross-fiber in-plane strain, *E*_rr_ is radial strain, and *E*_cr_, *E*_fr_ and *E*_fc_ are the shear components in their respective coordinate planes, C, b_1_, b_2_, and b_3_ are parameters to be chosen to fit experimental data. For simplicity, we set b_1_ = 0.8552, b_2_ = 1.7005, b_3_ = 0.7742 in Eq. (13) so that we can have a single parameter C for comparison. Figure 3 gave material stress-stretch curves for a baseline model with RVA pacemaker. The least-squares method was used to find the equivalent Young’s moduli (YM) for the material curves for easy comparison.

As patient-specific fiber orientation data was not available from these patients, we chose to construct a two-layer LV model and set fiber orientation angles using fiber angles given in Axel (2002). Fiber orientation angles were set at -60 degree and 80 degree for epicardium (outer layer) and endocardium (inner layer), respectively. Fiber orientation can be adjusted when patient-specific data becomes available (Tang et al., 2010).

### A Pre-shrink Process and Geometry-Fitting Technique for Mesh Generation

Under *in vivo* condition, ventricles are pressurized and the zero-stress ventricular geometries are unknown. In our model construction process, a pre-shrink process was applied to *in vivo* end-systolic ventricular geometries to generate the starting shape for the computational simulation (Fan et al., 2018). A geometry-fitting mesh generation technique was also used to generate mesh for our models (Tang et al., 2010). Mesh analysis was performed by decreasing mesh size by 10% (in each dimension) until solution differences were less than 2%. The mesh was then chosen for our simulations.

### Solution Methods and Data Collection for Statistical Analysis

The Echo-based anisotropic LV models were constructed for the four different pacing locations and the models were solved by ADINA (ADINA R&D, Watertown, MA, United States) using unstructured finite elements and the Newton-Raphson iteration method. The “Re-Start” feature in ADINA was used to adjust material parameters at each numerical time step to implement the potential conduction of myocardium. Flow velocity, flow shear stress (FSS) and stress/strain distributions were obtained for analysis. Because stress and strain are tensors, for simplicity, maximum principal stress (Stress-P_1_) and strain (Strain-P_1_) were used and referred to as stress and strain in this paper.

## Results

It is common to use selected cut-surfaces and critical time points (begin-filling, peak velocity during filling, begin-ejection, peak velocity during ejection, etc.) to demonstrate and compare solution behaviors. For our modeling set-up, the time points for begin-filling and end-ejection are connection points of systole and diastole phases. The same is true for end-filling and before-ejection time points. This explanation should be helpful to understand why we mainly used end-filling and end-ejection in our comparative analyses.

### Simulating Electrical Signal Conduction by Echo-Based Fluid-Structure Interactions Ventricle Model

**FIGURE 4:**
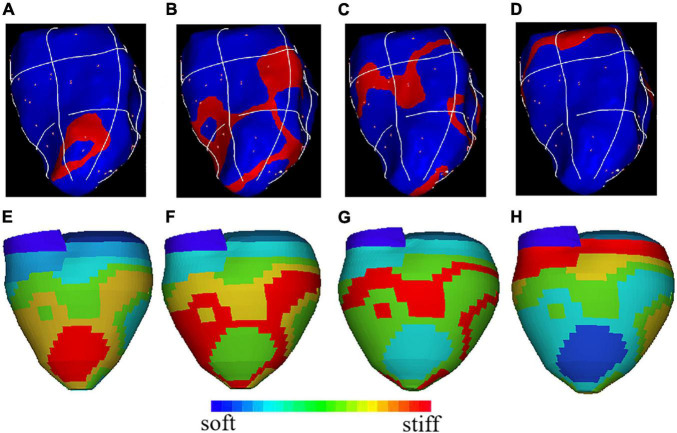
The electrical signal conduction diagrams with right ventricular apex (RVA) pacemaker at different phases and the corresponding 3D geometry model showing regional stiffness variation. **(A)** Electric signal mapping during Phase 1; **(B)**. Electric signal mapping during Phase 2; **(C)**. Electric signal mapping during Phase 3; **(D)**. Electric signal mapping during Phase 4; **(E)**. Model stiffness mapping corresponding to **(A)**; **(F)**. Model stiffness mapping corresponding to **(B)**; **(G)**. Model stiffness mapping corresponding to **(C)**; **(H)**. Model stiffness mapping corresponding to **(D)**.

The atrial and ventricular contraction and relaxation are necessary conditions for the heart to achieve blood pumping and promote blood circulation, and the excitatory process of the cell membrane is the initiating factor that triggers the contractile response. On the one hand, myocardium is functionally a syncytium, and the excitability generated in any part of the myocardial cell membrane can not only spread along the entire cell membrane, but can also be transmitted to other cardiomyocyte through the disc, thereby causing excitation and contraction of the whole myocardial. On the other hand, when the ventricular myocardium is excited, the action potential expands from the site of excitation to the periphery, thereby affecting myocardial contraction. In our model, the mapping between material stiffness and ventricular electrical signals is quantified based on data measured on pig implanted pacemakers, and the potential conduction of the myocardium is simulated by the softness of the material. Figure 4 shows the electrical signal conduction diagram and the corresponding 3D reconstruction geometry model at different phases. The red region in Figure 4E is the stiffest part of the model material, corresponding to the red region of the myocardial electrical signal conduction at this time in Figure 4A. And along the direction of electrical signal conduction, the myocardial material is getting softer. LV myocardial material parameter values at different phases are given in Table 2. The myocardial material was stiffer, C in Eq. (13), the mean YM value for the fiber direction (YM_f_) and the mean YM value for the circumferential direction of the fiber (YM_c_) were higher.

**TABLE 2:** Material parameters from Right ventricular apex (RVA) model at different phases.

	Phase 1			Phase 2		
Color	C(kPa)	YM_f_(kPa)	YM_c_(kPa)	C(kPa)	YM_f_(kPa)	YM_c_(kPa)
Red	21.65	622.4	215.2	18.76	539.4	186.5
Yellow	19.48	560.2	193.7	16.89	485.5	167.9
Green	17.32	497.9	172.2	15.01	431.5	149.2
Cyan	15.15	435.7	150.7	13.13	377.6	130.6
Blue	14.07	404.6	139.9	12.20	350.6	121.2
Darkblue	12.99	373.5	129.1	11.26	323.7	111.9

	**Phase 3**			**Phase 4**		
**Color**	**C(kPa)**	**YM_f_(kPa)**	**YM_c_(kPa)**	**C(kPa)**	**YM_f_(kPa)**	**YM_c_(kPa)**

Red	15.59	448.1	155.0	20.93	601.7	208.1
Yellow	13.85	398.4	137.7	18.83	541.5	187.2
Green	12.99	373.5	129.1	17.79	511.4	176.8
Cyan	12.12	348.6	120.5	16.74	481.3	166.4
Blue	11.26	323.7	111.9	14.65	421.2	145.6
Darkblue	10.39	298.8	103.3	12.56	361.0	124.8

*YM_f_: YM value for the fiber direction; YM_c_: YM value for circumferential direction of the fiber.*

### Right Ventricular Outflow Tract Pacing Has a Higher Maximum Velocity Value and Flow Shear Stress

**FIGURE 5:**
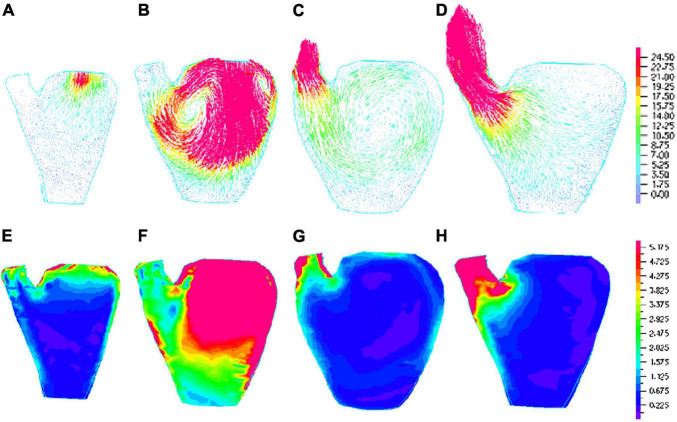
Flow velocity and flow shear stress (FSS) plots of right ventricular outflow tract (RVOT) pacemaker model. **(A)** Velocity vector plot at begin-filling, *t* = 1.28s, maximum velocity = 29.08cm/s, mitral valve opens, filling begins, aortic valve is closed; **(B)**. Velocity vector plot at the peak of filling, *t* = 1.56s, maximum velocity = 329.9cm/s, mitral valve is open, aortic valve is closed; **(C)**. Velocity vector plot at begin-ejection, *t* = 1.78s, aortic valve opens, ejection begins, mitral is closed, maximum velocity = 70.96cm/s; **(D)**. Velocity vector plot at *t* = 2.06s, maximum velocity = 164.9 cm/s, aortic valve is open, mitral valve is closed; **(E)**. FSS band plot at begin-filling, *t* = 1.28s, maximum = 10.87 dyn/cm^2^; **(F)**. FSS band plot at the peak of filling, *t* = 1.56s, maximum = 192.9dyn/cm^2^; **(G)**. FSS band plot at begin-ejection, *t* = 1.78s, maximum = 51.24dyn/cm^2^; **(H)**. FSS band plot at peak of ejection, *t* = 2.06s, maximum = 106.8dyn/cm^2^.

The flow dynamics index is an important indicator for characterizing and evaluating cardiac function under pacing. FSS reflects the influence of flow on LV inner surface and ventricle valves. Table 3 gives the maximum velocity values over the whole LV flow domain and the average FSS on LV inner surface at selected time points from the four models studied. Using the NP model as baseline, at the peak of filling, velocity magnitude for RVOT and PIVS pacing models were 13% and 5% higher than that of the NP, respectively. FSS for RVOT and PIVS pacing models were 45% and 30% higher than that of the NP, respectively. However, velocity magnitude and FSS for RVA were 4% and 9% lower than that of the NP, respectively. At the peak of ejection, velocity magnitude for RVOT and PIVS pacing models were 50% and 23% higher than that of the NP model, respectively. FSS for RVOT and PIVS pacing models were 44% and 36% higher than that of the NP, respectively. Moreover, RVOT pacing model has higher maximum velocity and FSS values. However, velocity magnitude and FSS for RVA were 6% and 6% lower than that of the NP, respectively. Figure 5 gave flow velocity and FSS plots from the RVOT model. It should be noted that global FSS maxima occurred near the mitral and aortic valve area, as expected.

**TABLE 3:** Velocity and flow shear stress (FSS) mean values at different times from 4 pacing models.

	Begin-filling		Peak of filling		Begin-ejection		Peak of ejection	
	Velocity (cm/s)	FSS (dyn/cm^2^)	Velocity (cm/s)	FSS (dyn/cm^2^)	Velocity (cm/s)	FSS (dyn/cm^2^)	Velocity (cm/s)	FSS (dyn/cm^2^)
NP	17.60	0.2142	293.1	4.572	59.87	2.796	109.9	3.359
RVA	17.01	0.1713	280.6	4.153	56.89	2.319	103.4	3.157
RVOT	29.08	0.4531	329.9	6.616	70.96	3.783	164.9	4.831
PIVS	22.51	0.3982	306.2	5.956	66.75	3.452	135.6	4.563

### Right Ventricular Outflow Tract Pacing Have Higher Stress and Strain

**FIGURE 6:**
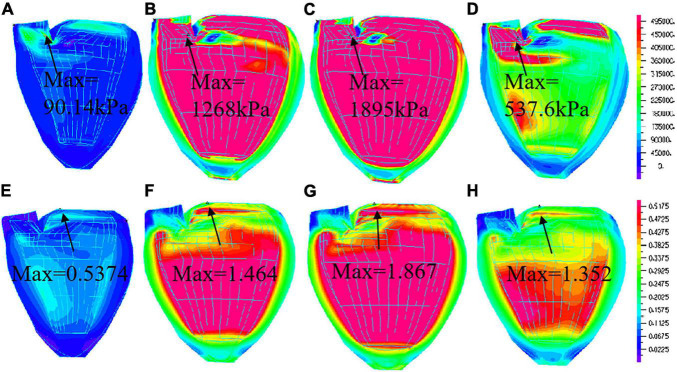
Stress and Strain plots of right ventricular outflow tract (RVOT) model. **(A)** Stress distribution at begin-filling; **(B)**. Stress distribution at the peak of filling; **(C)**. Stress distribution at begin-ejection; **(D)**. Stress distribution at peak of ejection; **(E)**. Strain distribution at begin-filling; **(F)**. Strain distribution at peak of filling; **(G)**. Strain distribution at begin-ejection; **(H)**. Strain distribution at peak of ejection.

Because of different pacemakers, different pacing methods or parts, the electrical impulses emitted are different for myocardial activation sequence, and the effects on myocardial motion will be different. Therefore, studying the movement of left ventricular myocardium under different pacing conditions is of positive significance for the evaluation of artificial pacing therapy. Ventricle stress and strain are good measure about how stiff ventricle muscle is. It is of interest to calculate LV stress/strain conditions for comparisons. Comparison of average stress and strain values on LV inner contours of four models were given in Table 4. Using NP model as baseline, at the peak of filling, stress of PVOT and PIVS pacing models were 18% and 10% higher than that of NP model, respectively. Moreover, Strain of PVOT and PIVS models were 13% and 5% higher than that of NP model, respectively. Stress of RVA pacing model was 4% lower than that of NP model. Strain of RVA model close to that of NP model. At the peak of ejection, stress of RVOT and PIVS models were 54% and 39% higher than that of NP model, respectively. Moreover, strain of RVOT and PIVS pacing models were 59% and 53% higher than that of NP model, respectively. However, stress and strain of RVA pacing model were 6% and 18% lower than those of NP model, respectively. Figure 6 gives the stress and strain distribution plots of RVOT model.

**TABLE 4:** Stress and strain mean values at different times from 4 pacing models.

	Begin-filling		Peak of filling		Begin-ejection		Peak of ejection	
	Stress (kPa)	Strain	Stress (kPa)	Strain	Stress (kPa)	Strain	Stress (kPa)	Strain
NP	2.779	0.0882	81.21	0.5756	135.9	0.6979	33.46	0.4214
RVA	2.705	0.0760	77.94	0.5717	103.2	0.6312	31.47	0.3446
RVOT	4.285	0.1685	95.85	0.6475	168.4	0.8691	51.54	0.6684
PIVS	3.754	0.1502	89.36	0.6046	145.7	0.7617	46.67	0.6447

## Discussion

Compared with other medical imaging methods, ultrasound medical imaging technology has many advantages. Currently, echocardiography is the main imaging modality for the assessment of LV structure and functions. Echocardiography does not have radiation and is a safe imaging mode. In most cases, it is non-invasive and will not cause harm to humans. In addition, echocardiography imaging equipment is more accessible and requires lower diagnostic costs. More importantly, echocardiography can image soft tissue and display the anatomy and real activity of the internal organs of the human body in real time. It has become the main means of cardiac function assessment and heart disease diagnosis in most major hospitals. Echocardiography can non-invasively examine any cross-sectional image of the heart and is often used to estimate left ventricular wall segment motion (Chamsi-Pasha et al., 2017). However, the use of echocardiography relies on the visual observation of the two-dimensional image by the examiner, and lacks quantitative description. The left ventricular wall function can only be estimated semi-quantitatively, and the local motion of the left ventricular wall cannot be observed and estimated. In recent years, two-dimensional ultrasound technology has been widely used to evaluate ventricular function, but the positioning of the image plane is still affected by the movement of the patient and the movement of the operator, and the analysis of ventricular function must rely on the assumption of cross-sectional information or ventricular geometry, so two-dimensional ultrasound has the limitations of its technology itself. In contrast, the three-dimensional model of the heart can not only provide clinical three-dimensional morphological information, but also provide an intuitive and effective judgment tool for determining the three-dimensional spatial location and pathological characteristics of the lesion. Therefore, the study of three-dimensional models of the heart has become a commonly used diagnostic tool in medical research for cardiovascular diseases.

Cardiac therapy is an important non-pharmacological treatment for patients with arrhythmia. However, while the cardiac pacing treatment saves the patient’s life, how to obtain better pacing therapy to improve the cardiac function status and quality of life for the patients is a major problem that needs to be solved urgently in clinical cardiovascular disease. The pumping process of the heart involves three kinds of physiological activities: first, myocardial excitation, electrical activity, including cell membrane depolarization-repolarization periodicity, forming an electrocardiogram cycle, the physiological function of cardiac electrical activity dominates the periodic rhythm of the heart, heart electrical activity triggers myocardial mechanical motion, called excitation-contraction coupling. Second, myocardial relaxation, mechanical movement, including muscle fiber contraction-diastolic periodicity, the formation of cardiac cycle, myocardial mechanical movement caused by changes in the distribution of blood flow in the heart chamber, thereby achieving cardiac pumping function. Third, the distribution of blood flow field in the heart chamber is a category of hemodynamics. The distribution of myocardial mechanical motion and heart chamber flow field can indirectly reflect the state of cardiac electrical expansion. These three kinds of cardiac physiological activities complement each other and constitute an important part of the heart pumping blood. To date, a number of active cardiac mechanics models have been proposed for different uses. The most important application is to explore the relationship between electrical signaling and mechanical motion in the heart. Clinical trials have found that asynchronous electrical activation can cause filling and pumping dysfunction (Lee et al., 2018). This finding suggests a biomechanical effect of changes in cardiac activation sequence. In order to better understand the relationship between the electrical activation mode of the ventricle and the local sequence of mechanical strain. This paper introduces an echo-based left ventricular FSI simulation model to achieve cardiac pumping, and simulates myocardial electrical signal transmission by realizing myocardial mechanical motion and cardiac flow distribution. In this study, we not only quantitatively analyze the hemodynamic parameters, but also the advantages and disadvantages of the hemodynamic effects, combined with the movement of the left ventricular myocardium, and evaluate the effects of different pacing modes on cardiac function. Ventricle remodeling, disease development, tissue regeneration, patient recovery after surgery and many other cell biological activities are closely associated with ventricle mechanical conditions (Pagani et al., 2021).

With the development of technology and research, more and more evidences show that ventricular electrical activation sequence and ventricular contraction synchronization are important factors affecting cardiac function. The closer to the physiological state of cardiac pacing, the less the effect on long-term cardiac function. Physiological pacing is not only to ensure the atrial-ventricular sequence, but also to maintain the synchrony of biventricular electro-mechanical activity. At present, many studies tend to think that the direct His bundle pacing has the best effect (Kronborg et al., 2014; Sharma et al., 2015). The direct His bundle pacing contraction activation sequence is consistent with the normal sinus rhythm. The earliest contraction is the upper part of the ventricular septum, which is rapidly transmitted from the ventricular septum to the apex. And the left and right ventricle free wall, the outer side spread, and finally stopped at the same time on both sides of the ventricular base. In theory, His bundle is the best ventricular pacing site. For many years electro-physicists have been studying direct His bundle pacing in order to obtain normal or near normal ventricular activation sequences. Kronborg et al. (2014) showed that His or para-His pacing preserves LV ejection fraction and mechanical synchrony compared with RV septal pacing in patient with atrioventricular block and may be a future pacing strategy to prevent pacing-induced heart failure in selected pacemaker patients. Sharma et al. (2015) assessed the safety, feasibility, and success rates of His-bundle pacing in unselected patients without the use of a mapping catheter or a backup RV lead as compared to RVA pacing. However, the direct His bundle pacing has limited the operation and application of this kind of surgery because of the difficulty in surgery operation and the long operation time, high pacing threshold, potential damage or blocking of His bundle. Because of the location of the right ventricular septum near the His bundle, the proximal end of the ventricular septum is first contracted and excited, and rapidly spread to the left and right ventricles and apex through the interventricular septum, so that the left and right ventricular contraction activation is basically synchronized. The electrical activation synchrony of the left and right ventricles ensures that the simultaneous mechanical contraction of the biventricular, the ejection fraction is improved, the left ventricular diastolic filling time is increased, the mitral regurgitation is reduced, and get better acute and long-term hemodynamic effects. Da Costa et al. (2013) provided a comprehensive overview of RVOT pacing. Singh et al. (2015) assessed LV function and dyssynchrony in patients with RVOT pacing and conventional RVA pacing using equilibrium radionuclide angiography. Their results indicated RVOT pacing may lead to better preservation of LV function on longer follow-up. Yao et al. (2013) evaluated contractile patterns in the circumferential direction in patients with idiopathic frequent premature ventricular complexes form RVOT. The present study also shows RVOT pacing have better blood flow and myocardial motion.

Several limitations should be acknowledged in our modeling study: (a) only one health animal’s data were used in this study. The pacemaker implant benefits may be different for each individual. A multi-animal or animal with cardiac arrhythmia study and should be conducted to help us draw more valid conclusions and further verify and confirm our findings; (b) ventricle valve mechanics was not included. Valve mechanics plays an important role. However, including it requires considerable more data (valve morphology and material properties) and it remains to be our future modeling effort; (c) local ventricle deformation imaging data (by particle tracking) was not included. We are in need of patient-specific data such as fiber orientation, sarcomere length contraction rate, regional material properties, etc. Lack of such *in vivo* data and model construction cost are also considerations. (d) active contraction and expansion were modeled by material stiffening and softening without adjusting zero-stress ventricle geometries. (e) Further research needs to be done into options for alternative pacing methods, such as RVOT pacing, PVIS pacing, and how they correlate with long-term clinical outcomes.

## Conclusion

Patient-specific models of the cardiovascular system are a promising approach to personalized cardiovascular medicine. FSI models provide complete mechanical analysis including both flow forces and structural stress/strain conditions and fluid structure interaction. Correct ventricle flow characteristics and stress/strain calculations are of fundamental importance for many cardiovascular researches where mechanical forces play a role in disease initiation, progression and treatment strategy selections. The existence of alternatives to existing leads and pacing methods may permit improvement in long-term outcomes with chronic pacemaker therapy while also making therapies such as synchronous pacing available to a wider array of patients with clinical situations. Direct comparison studies between pacing options will be needed to better understand the electromechanical associations and how these correlates with long-term morbidity, mortality, and quality of life. Studies concentrating on the therapeutic benefits of existing experimental therapies will also allow for the development of parameters that may permit correlation of findings during acute animal studies with long-term clinical outcomes. The clinical value of the present model can be further assessed by testing its ability to predict cardiac functional alterations during cardiac resynchronization therapy and ultimately to help optimize the therapeutic protocol. Here we have made a first step toward this goal by defining the baseline model of a patient with dyssynchronous heart failure.

## Data Availability Statement

The original contributions presented in the study are included in the article/supplementary material, further inquiries can be directed to the corresponding authors.

## Ethics Statement

The animal study was reviewed and approved by the Ethics Committee of the First Affiliated Hospital of Nanjing Medical University. Written informed consent was obtained from the owners for the participation of their animals in this study.

## Author Contributions

JY and DX were collected the data. LF, LW, and DT were done computational modelling and results analysis. LF and DT wrote the manuscript. All authors contributed to manuscript revision, read, and approved the submitted version.

## Conflict of Interest

The authors declare that the research was conducted in the absence of any commercial or financial relationships that could be construed as a potential conflict of interest.

## Publisher’s Note

All claims expressed in this article are solely those of the authors and do not necessarily represent those of their affiliated organizations, or those of the publisher, the editors and the reviewers. Any product that may be evaluated in this article, or claim that may be made by its manufacturer, is not guaranteed or endorsed by the publisher.
